# Clopidogrel Administration Impairs Neurovascular Unit Recovery and Exacerbates Amyloid Beta Accumulation in Aged Mice Post-Stroke

**DOI:** 10.3390/ijms27062547

**Published:** 2026-03-10

**Authors:** Marina Paul, Jonathan W. Paul, Madeleine Hinwood, Rebecca J. Hood, Kristy Martin, Mahmoud Abdolhoseini, Sarah J. Johnson, Michael Pollack, Michael Nilsson, Frederick R. Walker

**Affiliations:** 1 College of Health, Medicine and Wellbeing, University of Newcastle, Callaghan, NSW 2308, Australia; jonathan.paul@newcastle.edu.au (J.W.P.); madeleine.hinwood@newcastle.edu.au (M.H.); rebecca.hood@adelaide.edu.au (R.J.H.); kristy.martin@newcastle.edu.au (K.M.); michael.pollack@health.nsw.gov.au (M.P.); 2 Hunter Medical Research Institute, 1 Kookaburra Circuit, New Lambton Heights, NSW 2305, Australia; sarah.johnson@newcastle.edu.au (S.J.J.); michael.nilsson@newcastle.edu.au (M.N.); 3 Centre for Rehab Innovations, University of Newcastle, Callaghan, NSW 2308, Australia; 4 Discipline of Anatomy and Pathology, School of Biomedicine, Faculty of Health and Medical Sciences, University of Adelaide, Adelaide, SA 5005, Australia; 5 College of Engineering, Science and Environment, University of Newcastle, Callaghan, NSW 2308, Australia; mahmoud.abdolhoseini@newcastle.edu.au; 6 Department of Clinical Neuroscience, Institute of Neuroscience and Physiology, The Sahlgrenska Academy, University of Gothenburg, 405 30 Gothenburg, Sweden; 7 Rehabilitation Research Institute of Singapore (RRIS), Nanyang Technological University, Singapore 639798, Singapore

**Keywords:** stroke, microglia, clopidogrel, cortex, aged, vasculature

## Abstract

Clopidogrel has been the most commonly used therapy for preventing secondary cardiovascular events since 1997 by inhibiting the purinergic receptor P2Y, G-protein coupled, 12 protein receptor (P2RY12). P2RY12 is critical for microglia function in the brain, where it facilitates repair processes following injury. Under normal conditions, the blood-brain barrier (BBB) prevents peripheral drugs like clopidogrel from entering the brain. However, stroke-induced BBB disruption may allow clopidogrel to interfere with neural recovery by impairing microglia activity. Recently, we demonstrated that clopidogrel worsened cognitive outcomes in young mice after stroke. In this study, we examined the effects of clopidogrel on aged mice, focusing on survival, body weight, neurovascular changes, immune response, and amyloid beta accumulation. Aged male mice underwent photothrombotic stroke (or sham surgery) and received daily clopidogrel or control treatment for 14 days. On day 15, brain tissue was analyzed. Clopidogrel treatment significantly reduced survival and body weight, decreased vessel density, increased vascular permeability, altered microglia activity, and increased amyloid beta levels in the peri-infarct region. Notably, some of these effects were not observed in young mice. These results suggest that BBB disruption in stroke mice enables clopidogrel to enter the central nervous system, where it impairs microglia-mediated restoration of BBB integrity and promotes amyloid accumulation, factors that may contribute to worsened cognitive recovery. This study raises the possibility that clopidogrel may similarly cross the BBB in older stroke patients, impacting microglial function, and emphasizes the need for further research into its mechanisms of action.

## 1. Introduction

Globally, one in four people will experience a stroke, and every year, six and a half million people will die from stroke, while 25.7 million people will survive [1]. It is estimated that 11% of individuals will have a recurrence within a year of their first stroke and 26% within 5 years [2]. Clopidogrel, an anti-blood-clotting agent, is given to stroke survivors to prevent further cardiovascular events. Since its approval by the US Food and Drug Administration in 1997, clopidogrel has been prescribed to over 90 million patients worldwide [3], making it one of the most prescribed antiplatelet medications in the world. Clopidogrel exerts its antiplatelet activity via antagonism of the purinergic P2Y12 receptor (P2RY12) [4]. Although not widely known or considered during the clopidogrel antiplatelet clinical trials, P2RY12s are also expressed within the brain, exclusively on the fine cellular processes of microglia, which are projections extending from the cell body that enable surveillance of the microenvironment [5,6,7,8]. Microglia are versatile resident immune cells of the brain that play a critical role in learning and memory, as well as essential brain repair functions, including responding to stroke-induced tissue damage [9,10,11,12,13].

Under normal circumstances, the metabolites of P2RY12 inhibitors cannot cross the blood-brain barrier (BBB). The BBB is a physical barrier that prevents the entry of blood components, toxic macromolecules, as well as drugs or substances used for drug-like effects into the brain, thereby playing a role in maintaining the homeostasis of the central nervous system (CNS) [14]. Notably, one of the hallmarks of ischemic stroke is the disruption of the BBB [15,16]. This disruption permits the extravasation of blood-borne cells, chemicals, fluid, and drugs into the brain parenchyma due to increased paracellular and transcellular permeability [17]. In the case of P2RY12 inhibitors, this can suppress P2RY12-mediated microglial activation. Indeed, emerging animal studies strongly support this possibility [18,19,20]. Cserep et al. [18] demonstrated that P2RY12 inhibition with PSB0739 in a mouse stroke model abolished microglial responses, resulting in larger lesions. Similarly, Lou et al. [19] reported impaired microglial process movement toward vascular injury sites in clopidogrel-treated mice. Our prior work in young adult mice (10–14 weeks, equivalent to 20–30 human years) showed that clopidogrel administration post-stroke reduced survival, body weight, and cognitive recovery while increasing vascular leakage, altering microglial morphology, and reducing peri-infarct T-cell numbers [20]. Collectively, these findings suggest that compromised vascular integrity post-stroke provides a pathway for clopidogrel to reach the CNS, where it alters microglial structure and function, leading to impaired cognitive performance post-stroke.

Given that 63% of strokes occur in individuals aged 50–70 years, compared to only 16% in those aged 15–49 years [1], the effects of clopidogrel in older populations warrant investigation. This study examines the impact of short-term clopidogrel administration in 16–17-month-old mice (equivalent to >50 human years [21]), a model with greater translational relevance to the predominant stroke demographic. We hypothesize that post-stroke clopidogrel administration in aged mice will (i) impair physiological outcomes, including survival and body mass, (ii) disrupt the neurovascular unit, affecting blood vessel integrity, microglia, neurons, and immune cells, and (iii) promote the accumulation of the neurotoxic protein, amyloid beta (β). These findings aim to elucidate clopidogrel’s CNS effects in the context of stroke-induced BBB disruption and inform safer therapeutic strategies for stroke survivors.

## 2. Results

### 2.1. Short-Term Clopidogrel Administration Decreased Mouse Survival Rates and Body Weight

**Figure 1:**
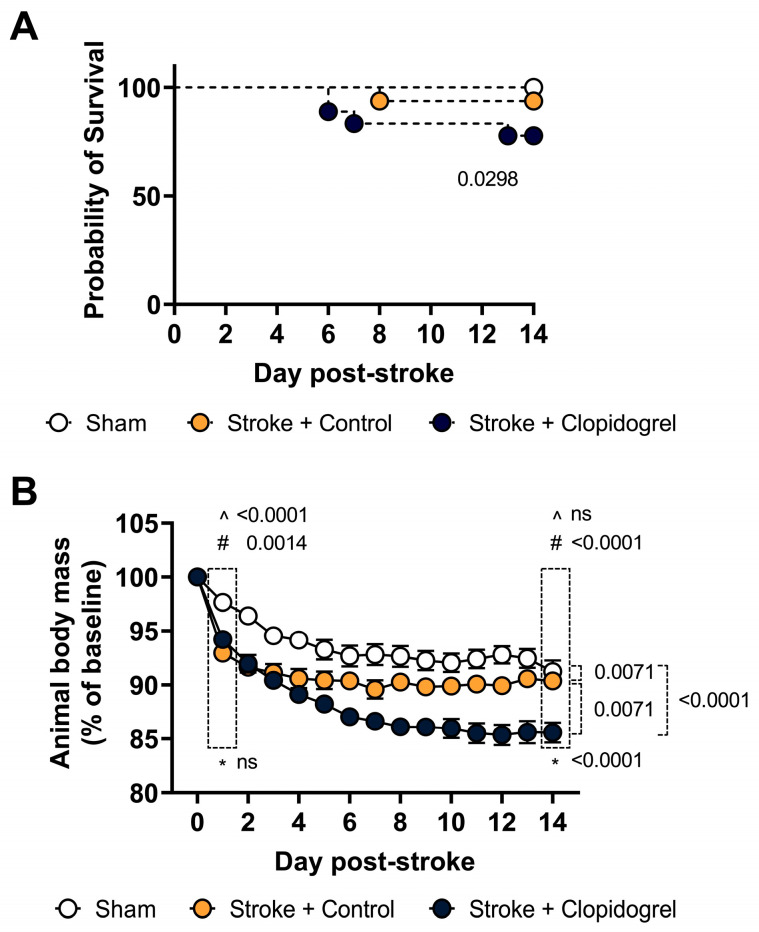
The effect of short-term clopidogrel administration on physiological parameters. (**A**) Survival rates and (**B**) body weights were recorded daily for 14 days post-stroke for sham-operated mice, stroke + control-treated mice, and stroke + clopidogrel-treated mice. Body weight change was calculated as a percentage (%) change from day 0. Mouse survival rates were analyzed by a simple survival analysis (Kaplan–Meier). Mouse body weight data were checked for normality using the Shapiro–Wilk normality test and then analyzed by two-way ANOVA with multiple comparisons (Benjamini, Krieger, and Yekutieli test). Data are mean ± SEM. Significant *p*-values are indicated. ns indicates not significant. ^ denotes changes between sham-operated and stroke + control animals, # denotes changes between sham-operated and stroke + clopidogrel animals, and * denotes changes between stroke + control and stroke + clopidogrel animals.

Mouse survival rates were recorded daily throughout the experiment. Although all mice were reared in the same environment following a photothrombotic stroke or sham surgery, 4 out of 18 mice from the stroke + clopidogrel treatment died during the 14 days of clopidogrel administration (77.78% survival rate; *p* = 0.0298), whereas 1 out of 16 mice from stroke + control-treated group died (93.75% survival rate; *p* = 0.0298) and 0 out of 16 mice from the sham-operated group died (100% survival rate; *p* = 0.0298) (Figure 1A).

Mouse body weight was recorded daily throughout the experiment. Overall body weight change was calculated as a percentage (%) change from baseline (day 0) to the last day of clopidogrel administration (day 14). Compared to sham-operated mice, there was a significant decrease in body mass of both stroke groups (control-treated, *p* = 0.0071; and clopidogrel-treated, *p* < 0.0001) across the 14 days. Additionally, there was a significant decrease in body mass of stroke mice treated with clopidogrel compared to control-treated (*p* = 0.0071) across the 14 days. Looking at the results more closely, on day 1 post-stroke, we recorded a significantly larger decrease in body mass in both the stroke + control-treated mice (*p* < 0.0001) and stroke + clopidogrel-treated mice (*p* = 0.0014) than in sham-operated mice; however, there was no significant difference in body mass between stroke + control-treated and stroke + clopidogrel-treated mice (*p* = 0.0867). By day 14 post-stroke, there was no significant difference in body mass between stroke + control-treated and sham-operated mice (*p* = 0.1433); however, the body mass of stroke + clopidogrel-treated mice remained significantly decreased compared to sham-operated mice (*p* < 0.0001). Moreover, by 14 days post-stroke, there was a significant decrease in body mass between stroke + control-treated mice and stroke + clopidogrel-treated mice (*p* < 0.0001) (Figure 1B).

### 2.2. Short-Term Clopidogrel Administration Altered Vasculature Post-Stroke

**Figure 2:**
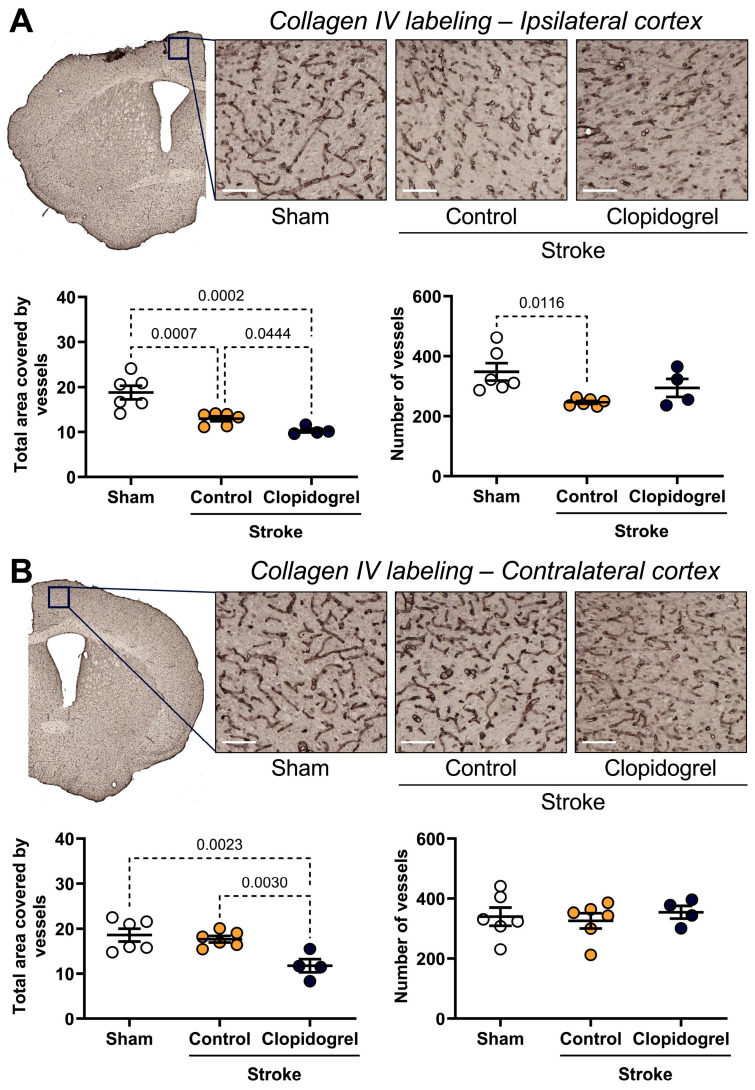
The effects of short-term clopidogrel treatment on vasculature within the ipsilateral and contralateral cortex. Collagen IV labeling within (**A**) the ipsilateral cortex region and (**B**) the contralateral cortex region (scale bar = 80 µm). The top images show the regions examined and representative collagen IV labeling observed for the three groups: sham, stroke + control, and stroke + clopidogrel. The graphs underneath show changes in the total area covered by vessels (left) and the number of vessels within the regions examined (right). Data were checked for normality using the Shapiro–Wilk normality test and then analyzed by two-way ANOVA with multiple comparisons (Benjamini, Krieger, and Yekutieli test). Data are mean ± SEM. Significant *p*-values are indicated.

Within the ipsilateral cortex, there was a significant decrease in the total area covered by collagen IV-labeled vessels in both stroke + control-treated mice (*p* = 0.0007) and stroke + clopidogrel-treated mice (*p* = 0.0002) compared to sham-operated mice. Moreover, there was a further significant decrease in the total area covered by collagen IV-labeled vessels in stroke + clopidogrel-treated mice (*p* = 0.0444) compared to stroke + control-treated mice. The number of collagen IV-labeled vessels detected was significantly reduced in stroke + control-treated mice (*p* = 0.0116), but not in stroke + clopidogrel-treated mice (*p* = 0.1266), compared to sham-operated mice. There was no difference in the number of collagen IV-labeled vessels between stroke + control-treated mice and stroke + clopidogrel-treated mice (*p* = 0.1266) (Figure 2A).

Within the contralateral cortex, there was a significant decrease in the total area covered by collagen IV-labeled vessels in the stroke + clopidogrel-treated mice compared to both sham-operated mice (*p* = 0.0023) and stroke + control-treated mice (*p* = 0.0030). There was no significant difference in the total area covered by collagen IV-labeled vessels between sham-operated aged mice and stroke + control-treated mice (*p* = 0.2041). There were no significant differences in the number of collagen IV-labeled vessels between the three groups (Figure 2B).

### 2.3. Short-Term Clopidogrel Administration Increased Vascular Leakage Post-Stroke

**Figure 3:**
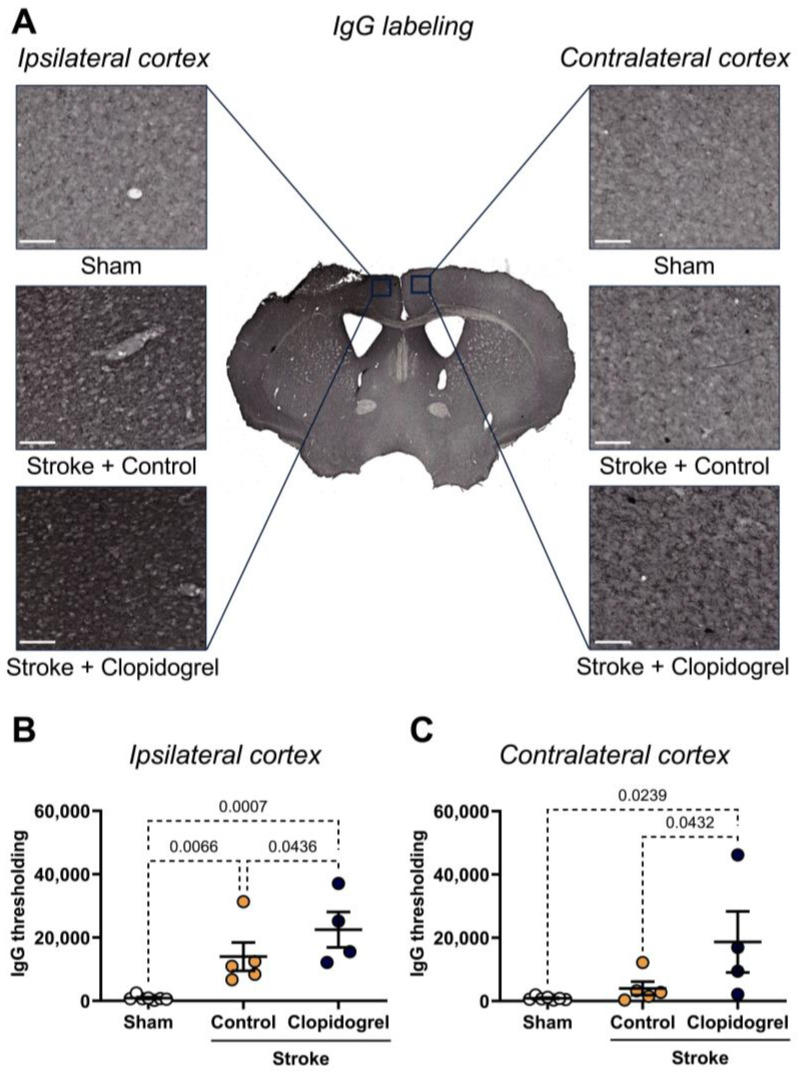
The effects of short-term clopidogrel treatment on vascular leakage in the ipsilateral and contralateral cortex. (**A**) Locations of the ipsilateral and contralateral cortex regions examined and representative labeling of IgG for the three groups: sham, stroke + control, and stroke + clopidogrel (scale bar = 80 µm). The graphs show the quantification of IgG thresholding within (**B**) the ipsilateral cortex and (**C**) the contralateral cortex. Data were checked for normality using the Shapiro–Wilk normality test and then analyzed by two-way ANOVA with multiple comparisons (Benjamini, Krieger, and Yekutieli test). Data are mean ± SEM. Significant *p*-values are indicated.

Within the ipsilateral cortex, there was a significant increase in IgG labeling of both stroke + control-treated mice (*p* = 0.0066) and stroke + clopidogrel-treated mice (*p* = 0.0007) compared to sham-operated mice. Additionally, IgG labeling in stroke + clopidogrel-treated mice was significantly elevated beyond that of stroke + control-treated mice (*p* = 0.0436) (Figure 3B).

Within the contralateral cortex, there was no significant change in IgG labeling between sham-operated mice and stroke + control-treated mice (*p* = 0.4115); however, IgG labeling in stroke + clopidogrel-treated mice was significantly elevated compared to both sham-operated mice (*p* = 0.0239) and stroke + control-treated mice (*p* = 0.0432) (Figure 3C).

### 2.4. Short-Term Clopidogrel Administration Altered Microglia Morphology Post-Stroke

**Figure 4:**
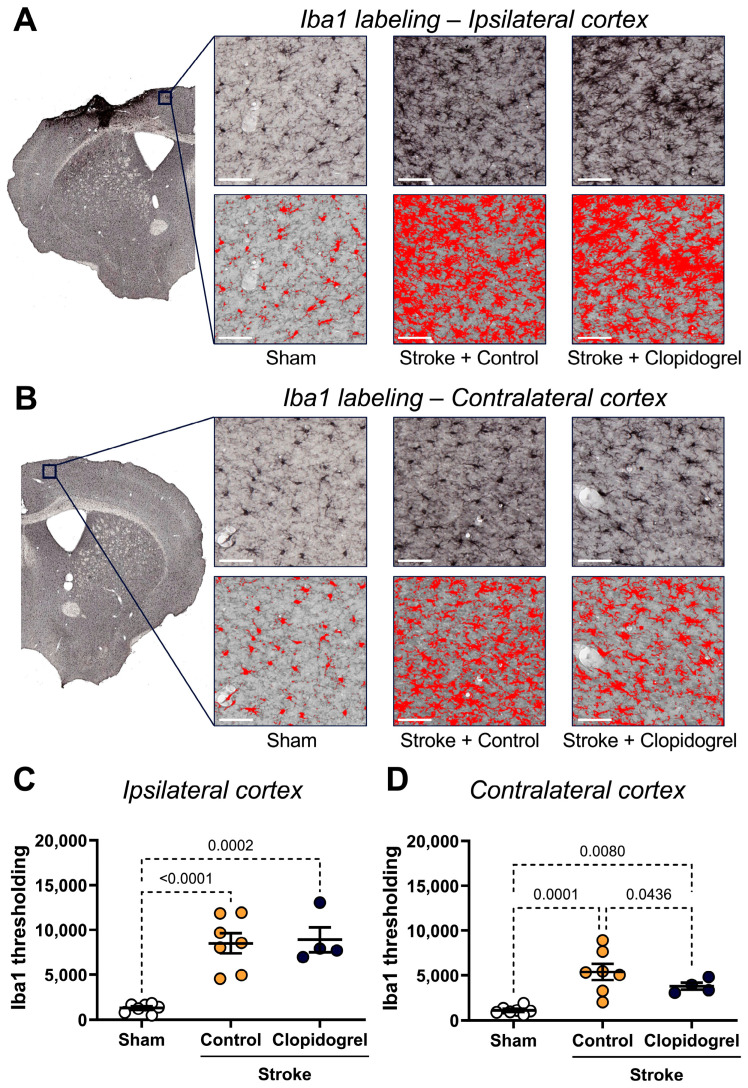
The effects of short-term clopidogrel treatment on microglia in the ipsilateral and contralateral cortex. The location of the ipsilateral (**A**) and contralateral (**B**) cortex regions examined and representative labeling of Iba1 for the three groups: sham, stroke + control, and stroke + clopidogrel (scale bar = 80 µm). The lower panels show material thresholded at the pixel intensity (PI) 103 (red). The bar graphs show the quantification of Iba1 thresholding within (**C**) the ipsilateral cortex and (**D**) the contralateral cortex. Data were checked for normality using the Shapiro–Wilk normality test and then analyzed by two-way ANOVA with multiple comparisons (Benjamini, Krieger, and Yekutieli test). Data are mean ± SEM. Significant *p*-values are indicated.

Within the ipsilateral cortex, there was a significant increase in Iba1 labeling of both stroke + control-treated mice (*p* < 0.0001) and stroke + clopidogrel-treated mice (*p* = 0.0002) compared to sham-operated mice. There was no difference in the Iba1 labeling between stroke + control-treated mice and stroke + clopidogrel-treated mice (*p* = 0.9569) (Figure 4C).

Within the contralateral cortex, there was a significant increase in Iba1 labeling in both stroke + control-treated mice (*p* = 0.0001) and stroke + clopidogrel-treated mice (*p* = 0.0080) compared to sham-operated mice. Additionally, Iba1 labeling in stroke + control-treated mice was significantly elevated beyond that of stroke + clopidogrel-treated mice (*p* = 0.0436) (Figure 4D).

**Figure 5:**
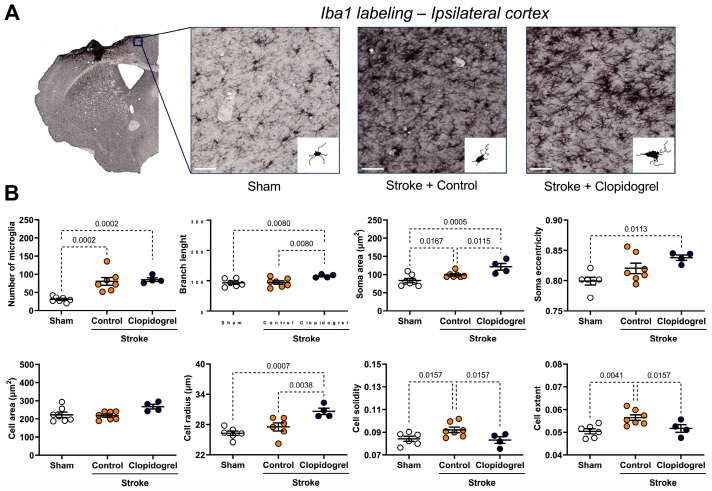
The effects of short-term clopidogrel treatment on microglia number and morphology within the ipsilateral cortex. (**A**) The top images show the regions examined and representative Iba1 labeling in the ipsilateral cortex across the three groups: sham, stroke + control, and stroke + clopidogrel (scale bar = 80 µm). Microglia were reconstructed based on MicroTrac software detection of Iba1 labeling, and then microglia number and morphology were analyzed. (**B**) The graphs underneath show changes in microglia number, branch length, soma area (µm^2^), soma eccentricity, cell area (µm^2^), cell radius (µm), cell solidity, and cell extent within the regions examined. All cells within one image were individually reconstructed and morphological parameters were averaged for each image, one image per animal. Data were checked for normality using the Shapiro–Wilk normality test and then analyzed by two-way ANOVA with multiple comparisons (Benjamini, Krieger, and Yekutieli test). Data are mean ± SEM. Significant p-values are indicated.

We further assessed the number and appearance of microglia within the ipsilateral cortex region using MicroTrac software (version 08b) (Figure 5A), which can quantify microglia number, soma area (µm^2^), soma eccentricity, total branch length, cell area (µm^2^), cell radius (µm), cell solidity, and cell extent. Compared to sham-operated mice, automated microglia quantification revealed that microglia in stroke + control-treated mice exhibited an increase in number (*p* = 0.0002), soma area (*p* = 0.0167), cell solidity (*p* = 0.0157), and cell extent (*p* = 0.0041) (Figure 5B). Quantification of microglia in stroke + clopidogrel-treated mice showed significant increases in microglia number (*p* = 0.0002), branch length (*p* = 0.0080), soma area (*p* = 0.0005), soma eccentricity (*p* = 0.0113), and cell radius (*p* = 0.0007) when compared to sham-operated mice (Figure 5B). Furthermore, compared to microglia in stroke + control-treated mice, microglia in stroke + clopidogrel-treated mice showed further significant increases in branch length (*p* = 0.0080), soma area (*p* = 0.0115), and cell radius (*p* = 0.0038). Microglia cell solidity (*p* = 0.0157) and cell extent (*p* = 0.0157) were significantly lower in stroke + clopidogrel-treated mice than in stroke + control-treated mice, but these parameters were not significantly different when compared to microglia from sham-operated mice (Figure 5B).

**Figure 6:**
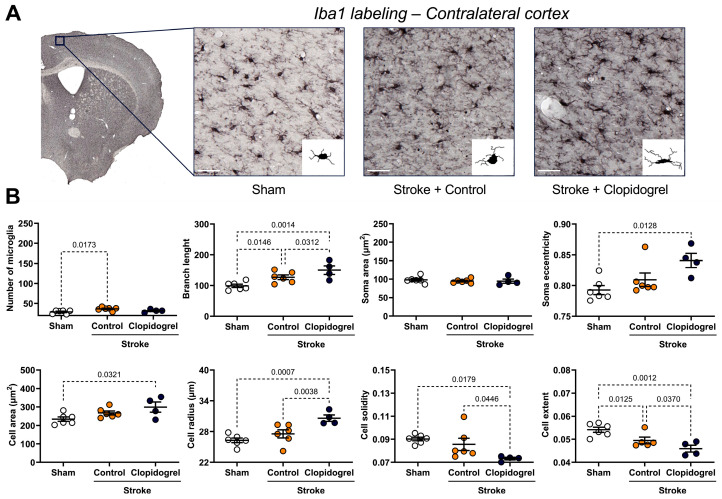
The effects of short-term clopidogrel treatment on microglia number and morphology within the contralateral cortex. (**A**) The top images show the regions examined and representative labeling observed for Iba1 within the contralateral cortex region for the three groups: sham, stroke + control, and stroke + clopidogrel (scale bar = 80 µm). Microglia were reconstructed based on MicroTrac software detection of Iba1 labeling, and then microglia number and morphology were analyzed. (**B**) The graphs underneath show changes in microglia number, branch length, soma area, soma eccentricity, cell area (µm^2^), cell radius (µm), cell solidity, and cell extent within the regions examined. All cells within one image were individually reconstructed and morphological parameters were averaged for each image, one per animal. Data were checked for normality using the Shapiro–Wilk normality test and then analyzed by two-way ANOVA with multiple comparisons (Benjamini, Krieger, and Yekutieli test). Data are mean ± SEM. Significant p-values are indicated.

Automated microglia detection also revealed changes in microglia morphology within the contralateral cortex (Figure 6A). Compared to sham-operated mice, microglia in stroke + control-treated mice showed significant increases in number (*p* = 0.0173) and branch length (*p* = 0.0146) and a significant decrease in cell extent (*p* = 0.0125) (Figure 6B). Compared to sham-operated mice, microglia in stroke + clopidogrel-treated mice showed significant increases in branch length (*p* = 0.0014), soma eccentricity (*p* = 0.0128), cell area (*p* = 0.0321), and cell radius (*p* = 0.0007), and significant decreases in cell solidity (*p* = 0.0179) and cell extent (*p* = 0.0012) (Figure 6B). Additionally, microglia in stroke + clopidogrel-treated mice showed significant further increases in branch length (*p* = 0.0312) and cell radius (*p* = 0.0038) as well as decreases in cell solidity (*p* = 0.0446) and cell extent (*p* = 0.0370) compared to microglia in stroke + control-treated mice (Figure 6B).

### 2.5. Short-Term Clopidogrel Administration Altered Neuronal Loss Post-Stroke

**Figure 7:**
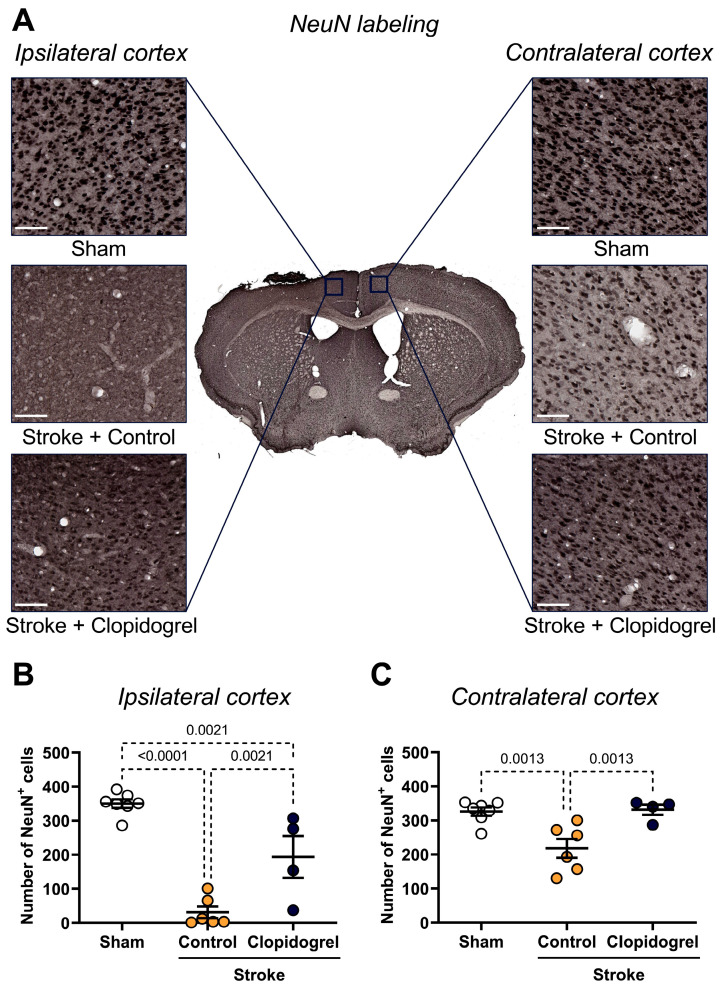
The effects of short-term clopidogrel treatment on neuron cell number in the ipsilateral and contralateral cortex. (**A**) The location of the ipsilateral and contralateral cortex regions examined and representative labeling of NeuN-positive cells for the three groups: sham, stroke + control, and stroke + clopidogrel (scale bar = 80 µm). The graphs show the quantification of NeuN-positive cells within (**B**) the ipsilateral cortex and (**C**) the contralateral cortex. Data were checked for normality using the Shapiro–Wilk normality test and then analyzed by two-way ANOVA with multiple comparisons (Benjamini, Krieger, and Yekutieli test). Data are mean ± SEM. Significant p-values are indicated.

Within the ipsilateral cortex, the number of NeuN-positive cells was significantly reduced in both stroke + control-treated mice (*p* < 0.0001) and stroke + clopidogrel-treated mice (*p* = 0.0021) compared to sham-operated mice. However, the decrease in the stroke + clopidogrel-treated mice was significantly less than the decrease in stroke + control-treated mice (*p* = 0.0021) (Figure 7B).

Within the contralateral cortex, there was a significant decrease in NeuN-positive cells in stroke + control-treated mice compared to both sham-operated mice (*p* = 0.0013) and stroke + clopidogrel-treated mice (*p* = 0.0013) (Figure 7C). There was no significant change in the number of NeuN-positive cells between sham-operated mice and stroke + clopidogrel-treated mice (*p* = 0.3001) (Figure 7C).

### 2.6. Short-Term Clopidogrel Administration Did Not Alter the Number of T Cells Post-Stroke

**Figure 8:**
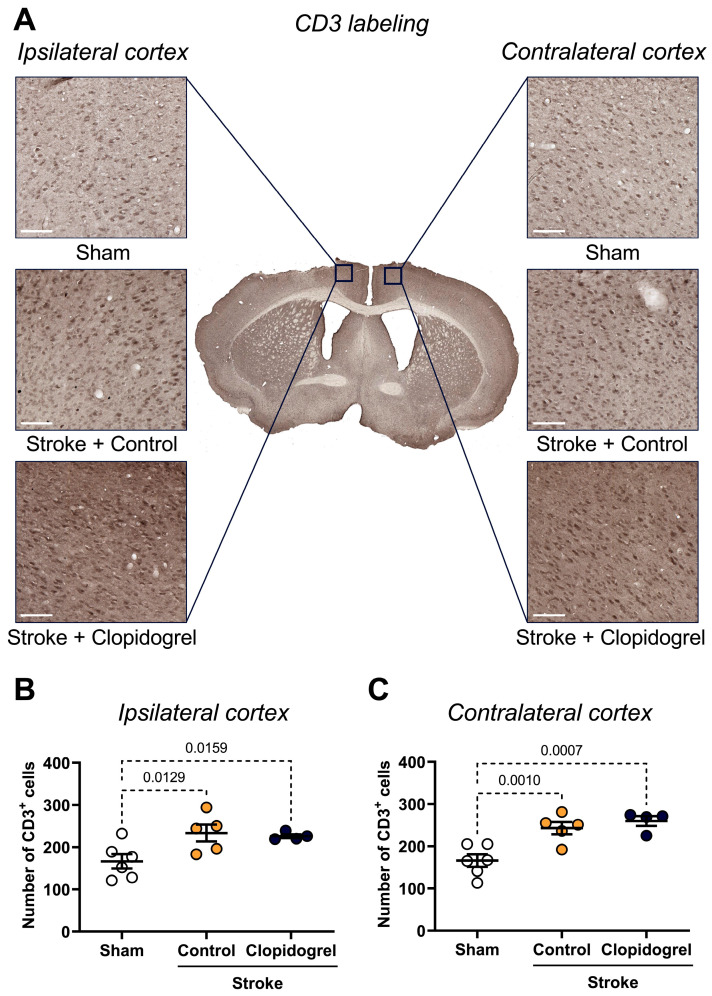
The effects of short-term clopidogrel treatment on T cell number within the ipsilateral and contralateral cortex. (**A**) The location of the ipsilateral and contralateral cortex regions examined and representative labeling of CD3-positive cells for the three groups: sham, stroke + control, and stroke + clopidogrel (scale bar = 80 µm). The graphs show the quantification of CD3-positive cells within (**B**) the ipsilateral cortex and (**C**) the contralateral cortex. Data were checked for normality using the Shapiro–Wilk normality test and then analyzed by two-way ANOVA with multiple comparisons (Benjamini, Krieger, and Yekutieli test). Data are mean ± SEM. Significant p-values are indicated.

Within the ipsilateral cortex, the number of CD3-positive cells was significantly elevated in both stroke + control-treated mice (*p* = 0.0129) and stroke + clopidogrel-treated mice (*p* = 0.0159) compared to sham-operated mice (Figure 8B). There was no difference in the number of CD3-positive cells between stroke + control-treated mice and stroke + clopidogrel-treated mice (*p* = 0.2709) (Figure 8B).

Similarly, within the contralateral cortex, there was a significant increase in the number of CD3-positive cells in both stroke + control-treated mice (*p* = 0.0010) and stroke + clopidogrel-treated mice (*p* = 0.0007) compared to sham-operated mice, but no difference between stroke + control-treated mice and stroke + clopidogrel-treated mice (*p* = 0.1583) (Figure 8C).

### 2.7. Short-Term Clopidogrel Administration Increased Amyloid Beta Accumulation Post-Stroke

**Figure 9:**
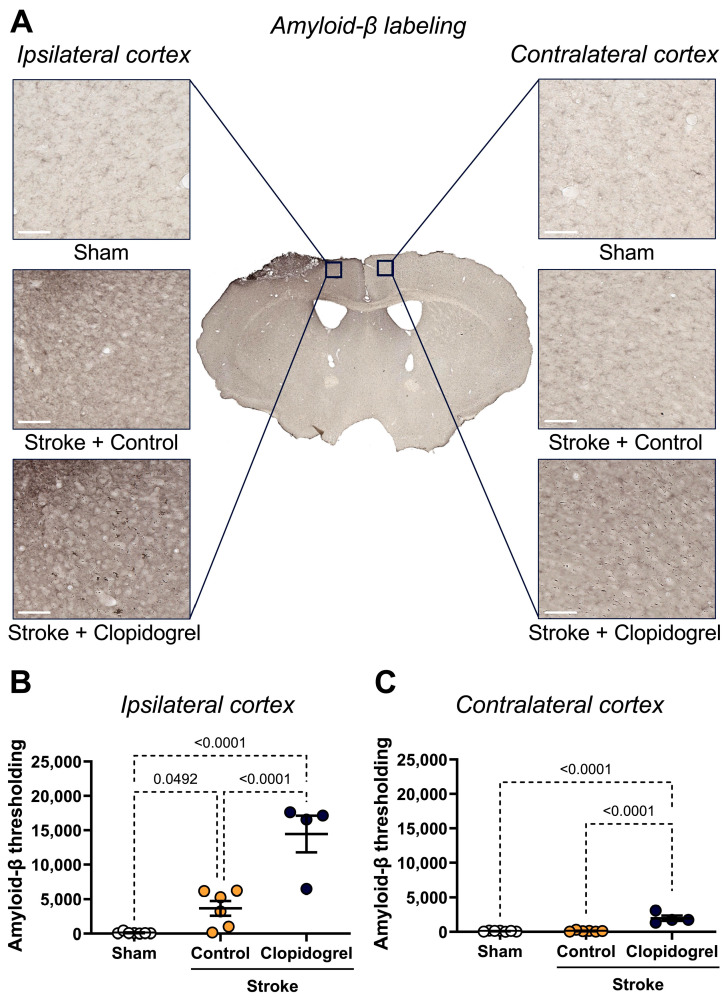
The effects of short-term clopidogrel treatment on amyloid-β accumulation in the ipsilateral and contralateral cortex. (**A**) The location of the ipsilateral and contralateral cortex regions examined and representative labeling of amyloid-β for the three groups: sham, stroke + control, and stroke + clopidogrel (scale bar = 80 µm). The graphs show the quantification of amyloid-β thresholding within (**B**) the ipsilateral cortex and (**C**) the contralateral cortex. Data were checked for normality using the Shapiro–Wilk normality test and then analyzed by two-way ANOVA with multiple comparisons (Benjamini, Krieger, and Yekutieli test). Data are mean ± SEM. Significant *p*-values are indicated.

Within the ipsilateral cortex, there was a significant increase in amyloid-β labeling of both stroke + control-treated mice (*p* = 0.0492) and stroke + clopidogrel-treated mice (*p* < 0.0001) compared to sham-operated mice. Additionally, amyloid-β labeling in stroke + clopidogrel-treated mice was significantly elevated beyond that of stroke + control-treated mice (*p* < 0.0001) (Figure 9B).

Within the contralateral cortex, there was no significant change in amyloid-β labeling between sham-operated mice and stroke + control-treated mice (*p* = 0.3213); however, amyloid-β labeling in stroke + clopidogrel-treated mice was significantly elevated compared to both sham-operated mice *(p* < 0.0001) and stroke + control-treated mice (*p* < 0.0001) (Figure 9C).

## 3. Discussion

Our study showed that in the aged cohort of mice, clopidogrel treatment significantly reduced mouse survival rate and body weight post-stroke, significantly increased vascular leakage and amyloid-β accumulation, and significantly altered the appearance of microglia within the peri-infarct region post-stroke. These data suggest that increased vascular permeability post-stroke may provide a pathway for clopidogrel to reach the CNS, potentially interfering with repair and recovery processes and impairing cognitive performance in aged mice. These findings are consistent with our earlier findings in young adult mice but appear to be more severe. Interestingly, in aged mice, we observed significant effects of clopidogrel in the contralateral cortex, including decreased blood vessel area, increased vascular leakage, altered microglial morphology, and increased amyloid-β, effects not previously observed in young mice. These effects in the contralateral cortex (away from the site of stroke) suggest that the aging-related breakdown of the BBB also permits the extravasation of clopidogrel.

Clinical trials indicate that the administration of clopidogrel is associated with an increased risk of bleeding complications in humans, ranging from minor to major events [22,23,24,25]. In this study, 22% of aged animals did not survive the 14 days of post-stroke clopidogrel administration (Figure 1A). Interestingly, only 11% of animals from our young cohort (prior study) did not survive the 14 days of post-stroke clopidogrel administration [20]. These results suggest that the risk of death doubles with age. While the precise reason why our young and aged mice died is yet to be determined, extra- or intracranial hemorrhage following repeated clopidogrel administration is a feasible possibility, considering outcomes from the previously mentioned trials.

Two common side effects of clopidogrel are altered or loss of taste and diarrhea. Precisely why clopidogrel causes loss of taste remains unknown. Nonetheless, patients who lose their sense of taste may also have decreased appetite, leading to weight loss. A phase IV clinical study of FDA data has shown that following clopidogrel treatment, 2.5% of patients (3528 people) experienced unintentional weight loss, while 4.06% (5727 people) experienced diarrhea. Moreover, the recent Clopidogrel Versus Aspirin in Patients at Risk of Ischemic Events (CAPRIE) trial showed that the incidence of diarrhea was significantly greater among clopidogrel-treated patients (4.5%) than among aspirin-treated patients (3.4%) [26]. Similarly, our aged mice had significantly greater initial post-stroke weight loss after clopidogrel treatment and did not regain weight over the 14-day study period, compared with control-treated mice (Figure 1B). This is different from findings in our young cohort of animals, where, after 14 days, the body weights of clopidogrel-treated stroke mice were not significantly less than the body weights of control-treated stroke mice [20]. These findings indicate that, in addition to aged mice being less able to recover from stroke/sham surgery (as none of the treatment groups regained weight), aged mice were also less able to recover/normalize from the effects of clopidogrel treatment. It would be interesting to see what happens to aged mice over a longer study period–is this the new normal weight for aged mice, or can they regain weight over time?

The BBB is a selectively permeable membrane that regulates the passage of a multitude of large and small molecules into the brain [14]. Structural disruption of endothelial cell junctions of the BBB and increased barrier permeability are pathological characteristics of both ischemic and hemorrhagic stroke in humans [15]. However, alterations and breakdown of functional components of the BBB have also been reported in the normal aging brain, even in the absence of underlying conditions [27]. Consistent with previous studies [20,28,29,30,31], a significant reduction in the total area covered by blood vessels was observed in the peri-infarct area post-stroke (Figure 2A). As collagen IV (the most abundant component of the basement membranes [32]) contributes to blood vessel integrity, stability, and functionality, these results suggest that stroke compromised the integrity of the BBB within the peri-infarct region, raising the possibility that blood-borne cells, chemicals, fluid, and drugs, such as clopidogrel, extravasate into brain parenchyma across the impaired BBB as a result of increased paracellular and transcellular permeability [17]. Indeed, consistent with previous studies [20,33], we detected significantly greater levels of IgG (primary immunoglobulin found in blood and extracellular fluid) labeling within the peri-infarct region post-stroke, suggesting extravasation of IgG into the peri-infarct region across a compromised BBB (Figure 3B). Interestingly, we found that clopidogrel treatment significantly exacerbated both blood vessel loss (total area covered by the blood vessels) (Figure 2) and extravascular leakage (IgG labeling) (Figure 3), indicating that clopidogrel did indeed enter the stroke-damaged brain, where it has interfered with brain cells essential for brain repair functions, such as microglia.

Microglia affect a remarkable variety of functions, including the regulation of synaptic plasticity associated with learning and memory, responding to CNS damage, and facilitating CNS repair [34]. Conditional removal or blockage of microglial activity after experimental stroke significantly exacerbated neuronal loss and increased infarct size [12,13]. This indicates that microglia play a vital role in brain repair and limit the extent of neurodegeneration after stroke. Microglia express high levels of P2RY12, which act as chemotactic receptors, directing microglial processes toward local sites of CNS injury and promoting the rapid closure of small openings in the BBB [5,6,19,35,36]. In the setting of vascular injury, clopidogrel suppressed the movement of juxtavascular microglial processes toward photolytic lesions in the vessel wall, which, in turn, suppressed the closure of BBB leaks, while the non-P2RY12-dependent platelet antagonist, acetylsalicylic acid (aspirin), did not reduce the motility of juxtavascular microglial processes toward injured capillaries [19]. As with our previous study in a young cohort of animals, our current study in an aged cohort of animals was designed to model a clinically relevant scenario, where an individual would experience a stroke and then, within 24 h, would commence clopidogrel administration. Clopidogrel treatment did not alter the number of microglia present within the peri-infarct region (Figure 5). This is not consistent with our previous results in the young cohort of animals, where clopidogrel-treated mice had significantly more microglia present within the peri-infarct region, indicating that post-stroke microglia moved to the site of the injury to facilitate repair, and then were immobilized and unable to vacate the peri-infarct region once clopidogrel crossed the stroke-damaged BBB [20]. The reason for this discrepancy could be that aged microglia are more responsive to stimulation, have altered morphology, and have a reduced ability to scavenge the brain parenchyma [37]. Moreover, aged microglia are less dynamic and ramified, whereas young microglia exhibit the opposite response, increasing their motility and becoming more ramified [38]. Moreover, in response to laser-induced focal tissue injury, aged microglia exhibited slower acute responses with a lower rate of process motility and cellular migration compared to young microglia [38]. Interestingly, in the longer post-injury term of 16 days, aged microglia demonstrated slower disaggregation from the injury site, indicating that responses of aged microglia, while slower to initiate, are more sustained [38]. Based on these findings, we believe that clopidogrel treatment did not alter the number of microglia due to either the slow but sustained response of aged microglia, or diminished expression of P2RY12 in aged microglia, or a combination of both phenomena. It would be interesting to ascertain whether differences in microglia numbers would emerge if the duration of our experiment were extended. Nevertheless, clopidogrel treatment affected multiple microglial parameters (process branch length, soma eccentricity, cell radius, cell solidity, and cell extent) in aged mice (Figure 5), whereas in our young mice, it significantly increased only soma area. This indicates that aged microglia are more severely impacted by clopidogrel treatment.

Stroke triggers a cascade of events that lead to rapid neuronal injury within the peri-infarct region. Initially, stressed neurons release damage-associated molecular patterns (DAMPs), which act as ‘eat-me’ signals attracting microglial phagocytosis. Microglia can rapidly phagocytose dead or dying neurons within hours post-stroke [39]. As expected, our results of NeuN labeling indicated that the number of neurons within the peri-infarct region was significantly reduced post-stroke (Figure 7B), consistent with our previous results indicating that microglia had phagocytosed the damaged neurons [20]. Interestingly, in this study of aged mice, clopidogrel significantly reduced the extent of neuronal loss post-stroke (Figure 7B). This could be attributed to aged microglia having a reduced ability to scavenge the brain parenchyma to remove neuronal debris, apoptotic bodies, and secreted proteins through phagocytosis [37] and thus, there was insufficient time for neurons to be phagocytosed before clopidogrel reached its therapeutic levels. It would, therefore, be interesting to perform more in-depth analyses of the condition of the neurons to ascertain whether clopidogrel allows stroke-damaged neurons to persist.

Following a stroke, the integrity of the BBB is compromised and cerebral microvessels become more permeable to molecules that are usually blocked from crossing the BBB [40]. This facilitates the immigration of systemic immune cells, including T cells, into the ischemic brain [40]. As expected, we observed a significant increase in the number of T cells within the peri-infarct region following photothrombotic stroke (Figure 8B). Moreover, unlike in our young cohort of animals [20], clopidogrel treatment did not decrease the number of T cells present within the peri-infarct region in our aged cohort of animals (Figure 8B). One interpretation is that even though clopidogrel enters the brain across the stroke-damaged BBB, aged microglia have a slow and sustained response; thus, we did not observe inhibition of T cell recruitment into the peri-infarct region.

The risk of cognitive impairment and dementia is 80% higher among stroke survivors, with the risk jumping to nearly 150% higher in those who had a bleeding stroke, compared to the general population [41]. Moreover, the risk of developing dementia was highest in the first year following a stroke, with stroke survivors facing a nearly three-fold increase in risk compared to their peers who did not have strokes [41]. The risk remained elevated 20 years after stroke compared to the general population [41]. There is currently little understanding of the mechanisms that lead to the development of dementia and cognitive impairment following stroke, although it appears that the pathophysiology is complicated and heterogeneous. Previous studies have shown that amyloid-β deposition occurs in the peri-infarct region [42,43], thalamus [44,45], and hippocampal regions [43,46] post-stroke and that such accumulation of neurotoxic proteins is highly associated with cognitive impairment in stroke patients [47]. Consistent with previous studies [42,43], amyloid-β deposition was observed in the peri-infarct region post-stroke (Figure 9B). Interestingly, following clopidogrel treatment, amyloid-β deposition was significantly exacerbated in the peri-infarct region (Figure 9B). Our group previously demonstrated that amyloid-β had scattered deposition in the brain parenchyma for four weeks post-stroke and at 12 weeks, was accumulated around the cerebral vessels in the peri-infarct area [43]. This is consistent with the findings of Howe et al. [48], who reported that stroke increased perivascular deposition of amyloid-β, and this effect was worsened in the aged cohort of animals. Moreover, this process was associated with both motor and cognitive impairment [48]. More recently, we found that the relatively short-term use of clopidogrel post-stroke negatively impaired cognition in young mice [20]. Although we did not use the paired-associate learning (PAL) task to test cognition in aged mice in the present study, our findings, taken together with previously published work, suggest the possibility of unanticipated cognitive effects associated with current platelet-directed strategies for preventing further cardiovascular events. As a follow-up, it would be interesting to examine cognition using the PAL task after clopidogrel treatment in an aged animal cohort.

Interestingly, significant changes were observed in the contralateral cortex following clopidogrel treatment, including decreased blood vessel area (Figure 2B), increased vascular leakage (Figure 3C), altered microglial morphology (Figure 6), and increased amyloid-β deposition (Figure 9C). These changes were not previously observed in the young cohort of animals [20]. This difference could be due to the breakdown of BBB functional components in the normal aging brain, allowing circulating clopidogrel to enter the brain, not only at sites where the BBB has been damaged by stroke but also at other sites within the brain. These results indicate that clopidogrel treatment has more severe consequences for aged mice.

Strengths of this study are the use of a clinically relevant scenario, where an individual would commence clopidogrel within 24 h following stroke; the use of aged mice, given that 63% of strokes occur in individuals aged 50–70 years; the use of photothrombotic occlusion model that produces an interruption of blood flow caused by platelet aggregation and alterations of the BBB, which are consistent with stroke in humans; the use of custom MATLAB software to analyze histological slides; and analysis of both contralateral and ipsilateral cortex. Limitations of this study is lack of assessment of cognitive impairment using the PAL task due to the limited number of aged mice; the lack of additional groups, such as sham + clopidogrel-treated mice and/or aspirin-treated group, due to the limited number of aged mice; further molecular characterization, such as mRNA and protein expression analysis, nevertheless study has been conducted, tissue collected, and the results will be published in the next manuscript; more detailed characterization of microglia; and further histological staining to determine whether the increased mortality is linked to hemorrhagic events.

In summary, our data demonstrate that in an aged cohort of animals, relatively short-term use of clopidogrel post-stroke doubled the risk of death and significantly exacerbated body weight loss, compared to clopidogrel’s effects in a young cohort of animals. Clopidogrel administration also significantly exacerbated the loss of total blood vessel area, an effect not previously observed in the young cohort of mice, and vascular leakage, as well as significantly affected multiple microglial parameters (process branch length, soma eccentricity, cell radius, cell solidity, and cell extent). Although we did not directly assess cognition in this study of aged mice, the fact that amyloid-β deposits have previously been linked to cognitive decline and that short-term clopidogrel treatment significantly increased amyloid-β deposition within both the ipsilateral and contralateral cortex regions, suggests that clopidogrel would likely have exacerbated cognitive decline in aged stroke mice. If such effects were carried over to humans, it would raise the prospect that clopidogrel administration may be hampering the cognitive recovery of aged stroke survivors. Indeed, this may explain why cognitive impairment is observed in stroke patients even after 20 years. While our studies identified a negative impact of clopidogrel on physiological changes, cognition, and changes in the neurovascular unit, others have reported neuroprotective benefits of clopidogrel administration [49]. However, the study was short in duration (48–72 h), with only three injections of clopidogrel administered, thus clopidogrel never achieved functional inhibition of P2RY12 *in vivo*. While further work is required, we believe our results highlight the possibility of unforeseen negative effects of current P2RY12-targeted platelet-directed strategies for secondary cardiovascular event prevention.

## 4. Materials and Methods

### 4.1. Animals

All experiments were approved by the University of Newcastle Animal Care and Ethics Committee (A-2013-340) and conducted in accordance with the New South Wales Animals Research Act and the Australian Code of Practice for the use of animals for scientific purposes. Male C57BL/6 mice were obtained from the Animal Services Unit at the University of Newcastle and aged until they were 16–17 months old (equivalent to >50 human years) [21]. Mice were maintained in a temperature (21.0 ± 1.0˚C) and humidity-controlled environment with food and water available *ad libitum*. Lighting was on a 12:12 h reverse light-dark cycle (lights on 19:00 h), with all procedures conducted in the dark phase. Mice were acclimatized to the environment for 7 days before initiation of the experiment.

### 4.2. Experimental Design

**Figure 10:**
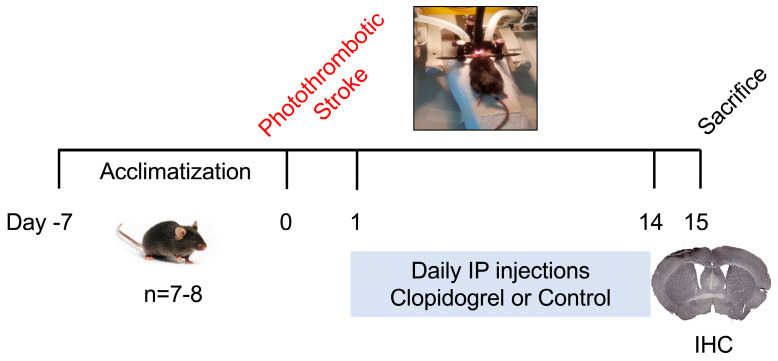
Experimental design timeline. Photothrombotic stroke or sham surgery was conducted on all mice. On day 1 post-stroke, mice were randomly treated with either clopidogrel or control daily for 14 days. On day 15, mice were euthanized, and brains were collected for immunohistochemistry analysis.

Following the acclimatization period, 50 mice were randomly allocated to one of three groups: sham (*n* = 16), stroke + control (*n* = 16), or stroke + clopidogrel (*n* = 18). The study was divided into two cohorts. The first cohort of mice (total of 23 animals: sham (*n* = 7), stroke + control (*n* = 8), or stroke + clopidogrel (*n* = 8)) was used to investigate histological analysis. The second cohort of mice (*n* = 27) was used for mRNA and protein expression analysis, and the results will be published in the next manuscript. This study was conducted and reported in accordance with the ARRIVE guidelines [50]. The experimental design is illustrated in Figure 10.

### 4.3. Photothrombotic Occlusion

Photothrombotic occlusion was performed as previously described [20,33,51,52,53,54,55]. Briefly, on day 0, mice were anesthetized (2% isoflurane and 2% oxygen) and injected intraperitoneally with either Rose Bengal (0.2 mL of 10 mg/mL; Sigma-Aldrich, Sydney, NSW, Australia) for photothrombotic stroke or sterile saline (0.2 mL of 0.9%; Pfizer, Sydney, NSW, Australia) for the sham procedure. After 8 min, the skull was illuminated for 15 min using a cold light source with a fiber optic end of 4.5 mm diameter positioned 2.2 mm left lateral of Bregma onto the exposed skull. Occlusion was directed into somatosensory and motor cortices (based on a Mouse Brain Atlas [56]). This photothrombotic occlusion model produces an interruption of blood flow caused by platelet aggregation and alterations of the BBB, which are consistent with stroke in humans [57]. One mouse from each group died overnight; a post-mortem revealed no apparent cause of death, but the death is conceivable to have occurred due to a stroke.

### 4.4. Clopidogrel Administration

On day 1, animals commenced daily intraperitoneal injections of clopidogrel (40 mg/kg; Selleck Chemicals, Houston, TX, USA) or control (25% dimethyl sulfoxide (DMSO; Sigma, Sydney, NSW, Australia) in 0.9% saline) [10,19,58,59].

### 4.5. Perfusion, Tissue Collection, and Tissue Processing

On day 15, animals were deeply anesthetized via intraperitoneal injection of sodium pentobarbital (0.2 mL of 325 mg/mL; Virbac, Milperra, NSW, Australia). Mice were perfused transcardially using ice-cold 0.9% saline followed by ice-cold 4% paraformaldehyde (PFA; Sigma, Sydney, NSW, Australia), pH 7.4. Brains were collected and post-fixed for four hours in 4% PFA, pH 7.4 at 4 °C. Brains were then transferred to a 12.5% sucrose (Chem Supply, Gillman, SA, Australia) solution in 0.1 M phosphate-buffered saline (PBS; Thermo Fisher Scientific, Thornton, NSW, Australia) and stored at 4 °C until sliced. Brains were sliced (coronal sections) using a freezing microtome (Leica, Sydney, NSW, Australia) at a thickness of 30 μm then kept in an anti-freeze solution (0.05 M PBS, sucrose, and ethylene glycol) at 4 °C. Fixed brain sections were stored at 4 °C until used for histological analyses.

### 4.6. Immunohistochemistry

For immunoperoxidase labeling, free-floating 30 µm PFA-fixed sections were immunostained using standard protocols, as previously described [20,33,51,52,53,54,55]. Briefly, sections were washed with 0.1 M PBS and endogenous peroxidases were quenched in 0.1 M PBS containing 3% hydrogen peroxide (Sigma, Sydney, NSW, Australia). Non-specific binding was blocked with 3% normal horse serum (Sigma, Sydney, NSW, Australia) for 30 min. Sections were then incubated in the presence of antibodies against collagen IV (CAT# ab6586; Abcam, Cambridge, UK; 1:1000; RRID: AB_305584), Iba1 (CAT#019-19741; WAKO, Richmond, VA, USA; 1:1000; RRID: AB_839504), NeuN (CAT#MAB377; Millipore, Burlington, MA, USA; 1:500; RRID: AB_2298772), CD3 (CAT#ab5690; Abcam, Cambridge, UK; 1:1000; RRID: AB_305055) or amyloid-β (CAT#SIG39320; BioLegend, San Diego, CA, USA; 1:500; RRID: AB_662798) with 2% normal horse serum for 72 h at 4 °C, washed 3 × 10 min with 0.1 M PBS and then incubated in goat anti-rabbit IgG (CAT#111-065-003; Jackson Immuno research laboratories, West Grove, PA, USA; 1:500; RRID: AB_2337959) or goat anti-mouse IgG (CAT#115-065-003; Jackson Immuno research laboratories; West Grove, PA, USA; 1:500; RRID: AB_2338557) for one hour at 25 °C. Following secondary antibody incubation, brain sections were washed for 3 × 10 min with 0.1 M PBS before final incubation in 0.1% extravadin peroxidase (Sigma, Sydney, NSW, Australia) for 90 min. Brain sections were washed 3 × 10 min with 0.1 M PBS and then immunolabeling was developed using a nickel-enhanced 3, 3′-diaminobenzidine (DAB; Sigma, Sydney, NSW, Australia) reaction. Tissues from all experimental groups were performed simultaneously, and the DAB reactions were developed for the same length of time following the addition of glucose oxidase (Sigma, Sydney, NSW, Australia; 1:1000). Sections were then washed, mounted onto chrome alum-coated slides, and cover-slipped.

### 4.7. Image Acquisition and Analysis

Images were taken at 40× with an Aperio AT2 using Aperio ImageScope (version 12.4; Leica, Wetzlar, Germany). For the IgG, Iba1, and amyloid-β analysis, we calculated the percentage of cumulative threshold material for the range of pixel intensity values (0–255) [20]. The pixel intensity level considered optimal for detecting genuine differences in immunoreactive signal was determined using ImageJ software (version 1.54g) to visualize the thresholding of cropped regions at individual pixel intensities. For collagen IV analysis, we determined the percentage area covered by the labeling and the number of vessels using ImageJ software (version 1.54g). For NeuN and CD3 analysis, we determined the number of positive cells using automated ImageJ software. For Iba1 morphological analysis, we used the ‘MicroTrac’ program written in MATLAB (version R2024b; Natick, MA, USA) [20,51,52,60,61]. This program is based on multilevel thresholding to identify cell soma, followed by a minimum spanning tree algorithm to trace the cell processes. The MicroTrac program quantitates the number of cells per image, as well as various morphological parameters, including soma area (µm^2^), soma eccentricity, number of primary branches, the total number of branch points, total branch length, cell area (µm^2^), cell solidity (cell area divided by the convex hull), and cell radius (µm, calculated from the center of the cell to the end of the longest branch). These metrics are calculated for each cell in an image and then averaged across images.

### 4.8. Statistical Analyses

All data were analyzed using GraphPad Prism v9.1.0 (San Diego, CA, USA) and are expressed as mean ± standard error of the mean (SEM). Data were checked for normality using the Shapiro–Wilk test. For multiple comparisons, a one-way analysis of variance (ANOVA) followed by a two-stage Benjamini, Krieger, and Yekutieli test for controlling the false discovery rate was used. For the mixed-effect analysis, a two-way analysis of variance (ANOVA) followed by a two-stage Benjamini, Krieger, and Yekutieli test for controlling the false discovery rate was used. For survival rates, a simple survival analysis (Kaplan–Meier) was performed. *p*-values ≤ 0.05 were considered statistically significant.

## Data Availability

The data are contained within this article.

## Abbreviations

The following abbreviations are used in this manuscript:

amyloid-β	amyloid-beta
ANOVA	analysis of variance
ARRIVE	animal research: reporting of *in vivo* experiments
BBB	blood-brain barrier
CAPRIE	clopidogrel versus aspirin in patients at risk of ischemic events
CD3	cluster of differentiation 3
CNS	central nervous system
DAB	3,3′-diaminobenzidine
DAMPs	damage-associated molecular patterns
DMSO	dimethyl sulfoxide
FDA	food and drug administration
mRNA	messenger ribonucleic acid
P2RY12	purinergic receptor P2Y, G-protein coupled, 12
PAL	paired-associate learning
PBS	phosphate-buffered saline
PFA	paraformaldehyde
SEM	standard error of the mean
